# The Landscape and Clinical Application of the Tumor Microenvironment in Gastroenteropancreatic Neuroendocrine Neoplasms

**DOI:** 10.3390/cancers14122911

**Published:** 2022-06-13

**Authors:** Shuaishuai Xu, Chanqi Ye, Ruyin Chen, Qiong Li, Jian Ruan

**Affiliations:** 1 Department of Medical Oncology, The First Affiliated Hospital, Zhejiang University School of Medicine, Hangzhou 310003, China; xushuaishuai@zju.edu.cn (S.X.); ycq7012@zju.edu.cn (C.Y.); chenruyin@zju.edu.cn (R.C.); liqiong0513@zju.edu.cn (Q.L.); 2 Key Laboratory of Cancer Prevention and Intervention, Ministry of Education, Hangzhou 310000, China

**Keywords:** gastroenteropancreatic neuroendocrine neoplasms, tumor microenvironment, angiogenesis, immunotherapy, combination therapy

## Abstract

**Simple Summary:**

The tumor microenvironment (TME) plays a role in promoting tumor progression. Elucidating the relationship between the TME and tumor cells will benefit current therapies. Therefore, this review summarizes the most recent relationship between the TME and tumor characteristics, discusses the differences in the TME at various sites along the digestive tract, and compares the TMEs of neuroendocrine tumors and neuroendocrine carcinomas. Microbial ecological changes in the TME were reviewed. The clinical application of the TME was summarized from bench to bedside. The TME can be used as a tumor drug target for diagnostic value, prognosis prediction, and efficacy evaluation, further revealing the potential of immune checkpoints combined with antiangiogenic drugs. The clinical application prospects of adoptive cell therapy and oncolytic viruses were described. The potential therapeutic approaches and strategies for gastrointestinal neuroendocrine neoplasms are considered.

**Abstract:**

Gastroenteropancreatic neuroendocrine neoplasms feature high heterogeneity. Neuroendocrine tumor cells are closely associated with the tumor microenvironment. Tumor-infiltrating immune cells are mutually educated by each other and by tumor cells. Immune cells have dual protumorigenic and antitumorigenic effects. The immune environment is conducive to the invasion and metastasis of the tumor; in turn, tumor cells can change the immune environment. These cells also form cytokines, immune checkpoint systems, and tertiary lymphoid structures to participate in the process of mutual adaptation. Additionally, the fibroblasts, vascular structure, and microbiota exhibit interactions with tumor cells. From bench to bedside, clinical practice related to the tumor microenvironment is also regarded as promising. Targeting immune components and angiogenic regulatory molecules has been shown to be effective. The clinical efficacy of immune checkpoint inhibitors, adoptive cell therapy, and oncolytic viruses remains to be further discussed in clinical trials. Moreover, combination therapy is feasible for advanced high-grade tumors. The regulation of the tumor microenvironment based on multiple omics results can suggest innovative therapeutic strategies to prevent tumors from succeeding in immune escape and to support antitumoral effects.

## 1. Introduction

Neuroendocrine neoplasms (NENs) are a type of malignant tumor that originates from neuroendocrine cells, and that is increasing in prevalence worldwide. It can occur in many different organs and tissues throughout the body, and the most frequent site of the disease is the gastrointestinal tract [1]. Moreover, it is also the second most common neoplasm of the digestive system [2]. NENs are divided into relatively well-differentiated neuroendocrine tumors (NETs) and comparatively poorly differentiated neuroendocrine carcinomas (NECs). The treatment scheme and prognosis of patients with NENs are mainly assessed by morphology and tumor grade, which are dependent on the mitotic count or Ki-67 labeling index, but they exhibit different survival outcomes, even when the same therapy is used for the same tumor grade. The foremost reason is the obvious heterogeneity of NENs, with some exhibiting inert, slow growth and some showing high metastasis [3]. Hence, more biological driver factors and more prognosis and treatment indicators should be elaborated.

Most drugs for NENs directly target tumor cells, and some drugs are hormone analogs [4]. However, the important role of the tumor microenvironment (TME) in neoplasm development and progression is being explored [5]. The TME encompasses all nontumor cells, such as immune cells, fibroblasts, vessel endothelial cells, and nerve-associated cells, as well as the extracellular matrix and soluble products, which contain collagen, fibronectin, chemokines, and other elements [6]. It is formulated as a critical hub for tumor heterogeneity, metastatic cascade initiation, clonal evolution, and therapeutic resistance [7]. Currently, drugs that are able to regulate the microenvironment show marvelous clinical therapeutic effects in many tumors. Immune checkpoint inhibitors, mainly cytotoxic T lymphocyte-associated protein 4 (CTLA-4), programmed cell death protein 1 (PD-1), and its ligand, PD-L1, are regarded as important immunotherapy treatments [8,9]. Drugs that affect neovascularization are beneficial for some malignancies. For NENs, the use of sunitinib and surufatinib as multiple-receptor tyrosine kinase inhibitors to influence neovascularization are approved for advanced NETs [10,11]. More clinical trials modulating the TME for NENs are underway. Moreover, different digestive organs show site-specific characteristics, such as changes in typical proteins, the proportion of disease subtypes, and survival time [12]. Hence, the mechanism by which the TME induces NEN tumor cells to express a broad functional spectrum and associated clinical application deserve further study in diverse gastrointestinal sites.

**Figure 1:**
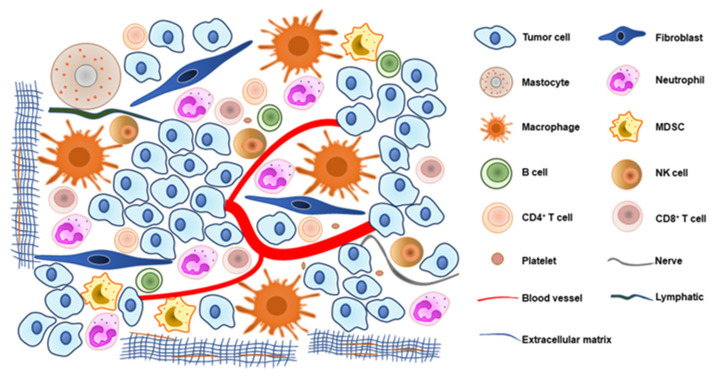
The tumor microenvironment atlas in gastroenteropancreatic neuroendocrine neoplasms.

We reviewed studies on the association between the TME and tumor cells in gastroenteropancreatic (GEP-) NENs (Figure 1), highlighting the recent research advances on regulating the TME to attenuate tumor growth and dissemination. We introduced each component in the TME and its corresponding clinical application and summarized the prospect of multi-drug combination therapy. More importantly, potential drug targets and innovative pharmaceutical technologies were provided for more therapeutic strategies for NEN management.

## 2. Innate Immune Cells

An increasing number of studies have pointed out the indispensability of innate immunity for tumor occurrence and development. Tumor cells can communicate directly with innate immune cells to form obvious immunosuppression and evade immune surveillance [13]. In addition, innate immunity is a mandatory prerequisite for boosting and recruiting adaptive immune cells to regulate antitumor immunity, and myeloid cell markers (CD33, CD163, and Arginase) are positively related to T cell markers for high-grade GEP-NENs, also revealing a potential linkage between myeloid cells and T cells [14].

Macrophages are an important component of the innate immune system and are characterized by high heterogeneity and plasticity. Studying the various functions of different macrophage subtypes in tumors to regulate immune system activity is conducive to uncovering the pathogenesis of diseases. The development and treatment of antitumor drugs that target macrophages are also the focus of current treatments [15]. In the majority of GEP-NENs, CD68^+^ macrophage infiltration can be detected [16]. It is more abundant in the metastatic foci in the liver than in primary lesions for insulinomas [17]. Single-cell sequencing of a pancreatic NET (G2) from the same patient [18] showed that macrophages could be divided into five clusters that are different from the classic M1 and M2 clusters. Among these clusters, cluster one is mainly located in the primary site, clusters two and five only exist in liver metastases, while chemokine CCL13 is only expressed in cluster one, and SPP1 is only present in clusters two and five. All these suggest that there are significant differences in the macrophages between the primary and metastatic sites. Additionally, the infiltration of CD68^+^ macrophages or CD163^+^ M2-polarized macrophages was associated with the high risk of recurrence as an independent disease-specific survival prognosis factor for postoperative patients with pancreatic NETs [19,20,21]. Notably, macrophage extracellular traps, as a form of macrophage death, could also predict recurrence prognosis in nonfunctional pancreatic NETs [22]. However, macrophages do not always reflect the outcomes of different treatments. Although CD163^+^ macrophages tend to increase in density and have an altered plump/epithelioid morphology after neoadjuvant peptide receptor radionuclide therapy, they are not related to progression-free survival in pancreatic NETs [23]. Microscopically, macrophages promote disease deterioration by facilitating the intravasation and extravasation of tumor cells, attracting angiogenesis and inhibiting antitumor immunity [24]. Macrophages can produce the cathepsin Z protease via the Arg-Gly-Asp motif to regulate integrin interactions and contribute to tumor invasion in pancreatic NETs [25]. When the recruitment and function of Tie2^high^ macrophages are decreased by rebastinib, the vascular permeability and liver metastasis of tumor cells can be inhibited in pancreatic NETs [26]. These malignant behaviors for tumor angiogenesis are mainly influenced by colony-stimulating factor-1, implying the possibility of colony-stimulating factor-1 as a drug target [17,27]. Nonetheless, there are not many drugs concerning macrophages in current clinical trials.

Neutrophils are also an important factor in tumor immunotyping and therapy evaluation, and the benign or malignant behavior and mechanism of neutrophils in NENs are worth exploring. The antitumor behavior of neutrophils is the manifestation of immune surveillance, but they also secrete a variety of factors to stimulate angiogenesis, degrade the extracellular matrix, and even promote tumor cell proliferation [28]. Tumor-infiltrating neutrophils were relatively lower than ten per 100 epithelial cells in pancreatic NETs [29], but they are an independent and unfavorable survival predictor [30]. Similar to macrophages, the presence of neutrophils is more common in metastatic lesions in the liver compared to in primary tumors, and their presence might be associated with an active complement pathway [31]. Neutrophil extracellular traps, which are a network of DNA, histones, and proteins released by activated neutrophils, are also terrible prognostic factors for recurrence in nonfunctional pancreatic NETs [22]. In addition, the neutrophil-dependent angiogenic switch is reinforced by HIF-1α, amplifying the Cyp46a1 enzyme and oxysterol 24S-HC in hypoxic areas of hyperplastic islets [32]. These results expound that neutrophils might enhance the immunosuppressive state of the TME and promote the proliferation and migration of tumor cells in NENs. Therefore, the precise regulation of the number and functional status of neutrophils is expected to be a tactic for prevention or treatment based on their contribution to the tumor progression of NENs.

Furthermore, increased tumor-infiltrating myeloid-derived suppressor cells in GEP-NENs are correlated with elevated metastasis [33]. Myeloid dendritic cells also demonstrate more infiltration in colorectal NETs than in NECs [34]. Notably, natural killer cells are not frequently observed at the metastatic sites of rectal NETs [35], so exploring the role of natural killer cells in metastasis is helpful in understanding their function. Mastocytes are obviously infiltrated in tumors and stromata, and suppressing their degranulation has been found to be useful for insulinoma therapy in mouse models [36]. Unfortunately, ibrutinib did not achieve an objective response in a phase II trial in GEP-NENs when used as a degranulation modulator [37]. Mastocyte infiltration was found as an independent predictor of prolonged progression-free survival and was found to be lower in patients with lymph node and distant metastasis than in patients without pancreatic NEN metastasis [38], so mastocyte infiltration might be a favorable factor, and more studies are needed to explore the role of mastocytes. As for other innate immune cells, very little has been explored in the NEN literature, but the roles of eosinophils, basophils, and dendritic cells are worth exploring. Their molecular and biological mechanisms might provide more potential therapeutic targets for diseases.

## 3. Adaptive Immune Cells

Tumor-infiltrating T cells and B cells are important components of the TME. CD8^+^ T cells are vital to cellular immunity. Killing tumor cells requires the activation of effective CD8^+^ T cells to produce a long-term antitumor immune response and more activated cytotoxic T cells [39]. CD4^+^ T cells are able to not only coordinate specific dendritic cells but also enhance the B cell immune response and assist cytotoxic T cells [40]. The number, location, and subgroups of tumor-infiltrating T cells and B cells demonstrate evident differences regarding survival endpoints.

In GEP-NENs, some research has pointed out that high tumor-infiltrating lymphocytes were associated with shorter survival and higher grade tumors [41]. In contrast, other studies found that high intratumoral CD3^+^ T cells exhibited better progression-free survival in GEP-NENs [42]. Tumor-infiltrating CD3^+^ T cells and CD8^+^ T cells might be associated with postoperative hepatic recurrence and overall survival in pancreatic NETs [43]. Therefore, the role of lymphocytes in predicting prognosis remains controversial. They are more frequently detected in the tumor stroma than in intratumoral regions and in NETs than NECs [44,45]. CD8^+^ T cells were more common in colorectal NETs than in NECs [34]. These results suggest that T cells are less common in intratumoral regions and in aggressive tumors. For tumor occurrence sites, CD3^+^ T cells, CD8^+^ T cells, and CD45RO^+^ T cells demonstrated more infiltration in pancreatic NETs than in small intestinal NETs [44]. CD4^+^ T cells show different prognostic values and drug sensitivity and have diverse subtypes. A higher Foxp3^+^ Tregs density was associated with poor overall survival in patients with pancreatic NETs [46]. The Th1 cytotoxic immune-phenotype response increased more than the Th2 response in intestinal NETs after lanreotide treatment [47]. Provided the importance of T cells, several bispecific antibodies targeting CD3 are currently in ongoing clinical trials. BI 764532, a delta-like ligand 3 and CD3 bispecific antibody, is currently in a phase one clinical trial for NETs with positive delta-like ligand 3 (NCT04429087). Another drug, XmAb^®^18087, a somatostatin receptor 2 and CD3 bispecific antibody, is in phase one clinical trials for progressive G1/2 NETs (NCT03411915). The clinical trials highlighting T cell-associated immunotherapy deserve more focus.

When it comes to B cells, CD20^+^ B cells are rare in pancreatic and ileal NETs [48]. In colorectal tumors, they demonstrate more infiltration in NETs than in NECs [34]. Nevertheless, they are infrequently observed at the metastatic sites of rectal NETs [35]. Hence, there are significant differences in the presence and content of B cells in tumors of diverse organs, and more clinical and biological research should be conducted to explore the potential miscellaneous function of B cells.

Tertiary lymphoid structures are a peritumoral ectopic immune cell aggregation area, but they are absent of intact cell surrounding structures such as the secondary lymphoid structure. In pancreatic NETs, the largest cell subpopulation of tertiary lymphoid structures is CD45RO^+^ T cells (39.35%) followed by CD20^+^ B cells (36.61%), CD4^+^ T cells (20.05%), CD8^+^ T cells (3.99%), and, rarely, CD68^+^ macrophages and Foxp3^+^ Tregs; additionally, CD20^+^ B cells are mainly situated in the follicular center, while diversified T cells overwhelmingly gather in parafollicular regions [49]. The presence of tertiary lymphoid structures is an independent advantageous prognostic factor for G1/2 pancreatic NETs [49]. The density, maturity, and distance (to the tumor) of tertiary lymphoid structures are also critical for assessing patient prognosis in other tumors [50,51]. Any treatment will affect the immune factors in the TME, including the tertiary lymphoid structures, so they may play an important role in the evaluation of the curative effect and enhancement of the antitumor effect, whereas there are relatively few studies on these directions in NENs, and they should be explored further.

## 4. Immune Checkpoints

**Figure 2:**
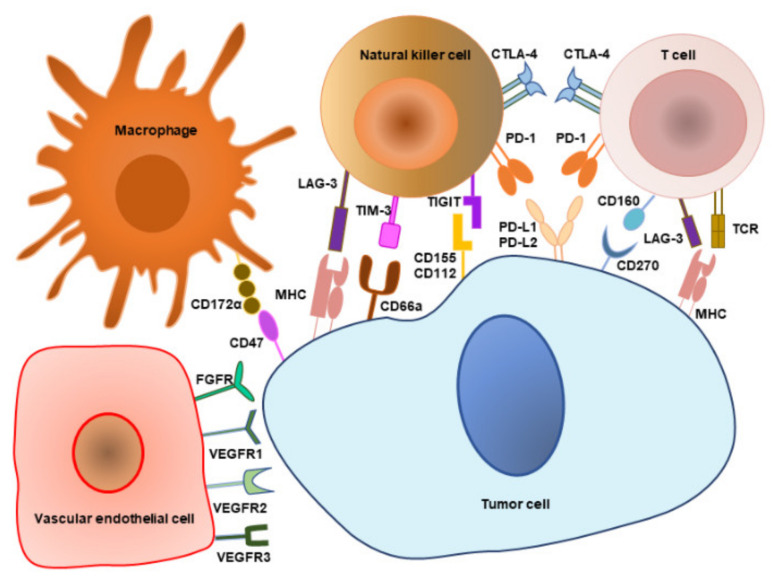
The interplay between tumor cells and tumor microenvironment cells. Tumor microenvironment cells encompass and interact with tumor cells. They express some molecules on the cell surface to regulate the occurrence and development of tumor, and these molecules are also common targets related to the tumor microenvironment. CTLA-4: cytotoxic T-lymphocyte-associated protein 4; FGFR: fibroblast growth factor receptor; LAG-3: lymphocyte activation gene 3; MHC: major histocompatibility complex; PD-1: programmed cell death protein 1; PD-L1: programmed death-ligand 1; PD-L2: programmed death-ligand 2; TCR: T cell receptor; TIGIT: T cell immunoreceptor with immunoglobulin and immunoreceptor tyrosine-based inhibitory motif domain; TIM3: T cell immunoglobulin and mucin domain-containing protein 3; VEGFR: vascular endothelial growth factor receptor.

Immune checkpoints are molecules that are produced by immune cells that regulate their own immune function and enable the immune system to remain within the normal range of activation without becoming over-activated. The abnormal expression and function of immune checkpoint molecules is one of the important reasons for tumor occurrence and progression. A checkpoint blockade is utilized to regulate dysfunctional antitumor immunity, and it has brought significant clinical benefits to many tumors, such as PD-1 inhibitors, PD-L1 inhibitors, and CTLA-4 inhibitors. Understanding the basic biological roles of these molecules in NENs is essential for the rational development of novel immune checkpoint blocking therapies (Figure 2).

In GEP-NEN patients, the PD-L1-negative region is dominant in tumor centers, while PD-L1 is mainly expressed in the tumors and immune cells at the frontier of tumor invasion, and the density of CD8 and PD-1 immune cell infiltration is higher in PD-L1-positive tumor regions [52]. High e PD-1 expression can distinguish worse survival and an elevated tumor grade in GEP-NENs [41], and approximately 16% of patients express PD-1 in GEP-NECs [53]. Moreover, PD-L1 expression is less than one-third or even sometimes 1% with weak-to-moderate intensity in GEP-NENs, and they also mainly occur in high-grade tumors [54] and in NECs [55,56]. In small intestinal NETs, PD-L1 positivity is relatively lower than in jejunal/ileal NETs, and lymph node spread is associated with a tumor cell rate with over 50% PD-L1 expression [57]. However, PD-1 and PD-L1 expression is low in metastatic sites of rectal NETs [35]. Regarding the overexpression mechanism of PD-L1, in addition to lymphocyte stimulation, variations in the copy number of PD-L1 genes might make a contribution to gastric NECs [58].

Moreover, microsatellite instability (MSI) and tumor mutational burden (TMB) are predictable drug response indicators for immunotherapy. Although a higher status of these two indicators was similarly more prevalent in high-grade tumors than in low-grade tumors in GEP-NENs, a higher MSI and TMB were only found in 4% and 7% of high-grade tumors, respectively (the threshold for a higher MSI was determined to be insertion or deletion at 46 or more loci through next-generation sequencing) [54]. Compared to G3 NETs, a higher MSI is only found in NEC cases (a higher MSI is determined by an overall MSI score based on the analyses of 95 loci through next-generation sequencing) [59]. MSI is frequent in colorectal NECs but is uncommon in other GEP-NENs (a high MSI was defined as being when at least two of five microsatellite loci showed instability via a fluorescent PCR-based assay) [60]. Moreover, patients with GEP-NECs had a median of 5.68 mutations/Mb for TMB but without a high MSI status (a higher MSI status was defined as when more than two software programs such as mSINGS, MSIsensor, and MSIseq were used) [61]. Pancreatic NETs had a 1.3% higher TMB rate and no high MSI status (the threshold for a higher MSI was determined as insertion or deletion at 46 or more loci through next-generation sequencing) [62]. These results suggest that a lower proportion of patients could be sensitive to associated immunotherapy. The differences in the high MSI results may be due to the lack of clear and uniform standards and may also be related to the site of the disease and testing methods. Fortunately, targeted therapy for another PD-1 ligand, PD-L2, might be considered in clinical practice, as the expression of PD-L2 is more pronounced compared to the rare expression of PD-1 or PD-L1 in small intestinal or pancreatic NETs [44,63].

Immune checkpoint inhibitors are also included in clinical trials. The anti-PD-1 agent toripalimab showed safe and beneficial activity for patients with first-line therapy failure, especially those with positive PD-L1 expression, high TMB, or high MSI status (NCT03167853) [64]. Another anti-PD-1 blockade, pembrolizumab monotherapy, was demonstrated to be able to be administered safely but with limited activity in patients with differentiated NENs (NCT02628067, NCT02267967) [65,66]. Nevertheless, after pembrolizumab treatment in high-grade NENs, CD4^+^ T cells and PD-1 expression were decreased, while T cell immunoreceptor with immunoglobulin and immunoreceptor tyrosine-based inhibitory motif domain expression and CD62L^−^ effector T cells were shown to be increased in peripheral blood tests [67]. These results reveal potential drug resistance but also suggest that other immune checkpoint inhibitors might ameliorate antitumor immunity when followed by or combined with one inhibitor. On the other hand, combinatorial immune checkpoint therapies are required to guide clinical improvement [68]. The anti-PD-1 blockade nivolumab and anti-CTLA-4 blockade ipilimumab doublet therapeutic schedule provided an evidently higher objective response rate (ORR): 44% for nonpancreatic NECs [69], 43% for high-grade pancreatic NENs [70], and 26% for high-grade and microsatellite-stable NENs [71]. Ipilimumab and nivolumab regimens also showed a 14.7% ORR in high-grade progressive NENs with pretreated cytotoxic chemotherapy [72]. Although immunotherapy has been widely reported to be a powerful therapeutic weapon for other tumor types, its success in GEP-NENs is not completely clear, with only a few clinical trials showing limited therapeutic activity [73]. More immune checkpoint-associated immunotherapy clinical trials are underway (Table 1).

On the other hand, novel tumor immune checkpoints are constantly being discovered and replenished [74,75], and some new immune checkpoints have also been detected in NENs, even in specimens treated with some immune checkpoint inhibitors [67]. Currently, more immune checkpoints are being elaborated upon in tumor studies, such as the B7 family, T cell immunoglobulin, and mucin domain-containing protein 3 (TIM-3), CD47, and CD74. HERV-H LTR-associating protein 2 and B7 family member H4, as two B7 family immune checkpoints, are richer in GEP-NETs and are associated with a high tumor grade and lymph node metastasis rate [76]. Additionally, CD47 and CD74 are highly abundant, but PD1, PD-L1, and TIM-3 are lacking in ileal NENs [45]. Tumor cells express CD47 to recede from macrophage engulfment [77], and low CD47 expression is favorable for tumor progression in pancreatic NETs [78]. In pancreatic NETs, immunosuppressive genes, including PD-L1, PD-L2, C10orf54, lymphocyte-activation gene 3, and indoleamine 2,3-dioxygenase 1, are rich in metastasis-like primary-1 subtype classification (accounting for 26–31% of patients) for T cell and M1 macrophage modulation [79]. The expression of another immune checkpoint, CD73, is significantly correlated with PD-L1 expression and is more highly detected in NECs than in NETs [80]. The CD73 inhibitor attenuates the malignant biological properties of pancreatic NET cancer stem cells [81]. The co-expression of PD-1/ICOS and PD-1/CTLA-4 is significantly higher than that of normal tissues in small intestinal NETs [82]. These results suggest the possibility of multi-target therapy with immune checkpoint inhibitors or two-drug combination therapy. These novel immune checkpoint inhibitors can be considered for further testing in clinical trials, and more immune checkpoints need to be disclosed. All of these findings imply that further detection, diagnosis, medication, and retesting after drug resistance of immune checkpoints should be explored in NENs.

## 5. Vasculature and Lymphatic Factors

New blood vessels are needed to supply nutrients and remove metabolites to support the growing tumor, and their formation is an important step in tumor progression and metastasis. The management of angiogenesis is not only influenced by the regulatory factors secreted by the blood vessels themselves but is also promoted by tumor cells. Other cells in the TME can also be involved in this process, such as tumor-associated macrophages, fibroblasts, and mastocytes (Figure 1).

Vascular endothelial-derived growth factor (VEGF) is critical in vasculogenesis and angiogenesis that mainly involves vascular permeability and neovascularization [83]. Of note, tumors with low VEGF protein expression are more aggressive than those with high VEGF expression, and VEGF expression is negatively connected with Ki-67 expression in GEP-NENs [84]. It is worth investigating the specific mechanism further. Many vascular factors participate in the VEGF/VEGFR pathways in the NEN mechanism (Figure 2). Elevated CDK5RAP3 facilitates angiogenesis via the AKT/HIF-1α/VEGFA signaling pathway in gastric NECs [85]. Neuropilin 2, a VEGFR2 coreceptor, can augment angiogenesis via the slingshot-1/cofilin/actin axis to induce tumor growth in pancreatic NETs [86]. Another study on the VEGFR family indicated that the extremely low expression of VEGFR2 and elevated VEGFR1 and VEGFR3 expression are likely to be observed in GEP-NENs and can be observed in 80% of pancreatic tumors [87]. Therefore, the biological and clinical role of neuropilin 2 should be studied further in NETs due to its low VEGFR2 expression. Furthermore, these vasculature factors also influence other cells and molecules in the TME. Semaphorin 4D, which takes advantage of platelet-derived growth factor B to modify pericyte coverage, induces and recruits macrophages in invasive tumor fronts to secrete stromal cell-derived factor 1, thus communicating with tumor cells in which CXCR4 promotes tumor invasion and metastasis in pancreatic NETs [88]. Periostin, which regulates VEGFA and fibroblast growth factor 2-adaptive alterations for revascularization, is related to M2-like macrophages and exhausts macrophages under a colony-stimulating factor one receptor antibody in pancreatic NETs, revealing how VEGFA might have an immunosuppressive function in addition to angiogenic activity [89]. Platelet-derived growth factor receptor alpha, which can upregulate VEGF to mediate tumoral vascular networks, is mainly rich in G2 patients and in G3 patients with mixed insular-acinar conditions in GEP-NENs [90]. Angiopoietin-2 promotes angiogenesis and vascular instability and increases vascular permeability and inflammation. It shows higher expression in patients with progressive GEP-NETs [91], and the dual angiopoietin-2/VEGFR2 blockade inhibits revascularization and tumor progression in VEGFR2-resistant pancreatic NET mice models [92]. This indicates the prospect of applying angiopoietin-2 inhibitors.

Many studies on anti-VEGF or anti-VEGFR therapies have shown that these factors can effectively inhibit angiogenesis and tumor growth in preclinical models. When used as anti-VEGF inhibitors, aflibercept and bevacizumab depict antitumoral activity, repressing tumor progression in colon NECs [93]. Therefore, the inhibition of the VEGF and VEGFR pathways has been identified as an important and effective antitumor mode in clinical practice. Sunitinib and surufatinib, multiple-receptor tyrosine kinase inhibitors that influence VEGFR, are beneficial and approved for advanced pancreatic NETs [11,94] and nonpancreatic NETs [10]. Another multiple-receptor tyrosine kinase inhibitor lenvatinib displayed an overall response rate of about 30% in patients with G1/2 GEP-NETs who had progressed after prior treatment [95]. As shown in Table 2, there are many ongoing clinical trials on other protein–tyrosine kinase inhibitors. Another therapeutic approach to vascular regulation is targeted vasculogenic mimicry, and an associated drug, CVM-1118, is also undergoing clinical trials for progressive G1/2 NETs (NCT03600233).

Lymphatic endothelial cells are involved in lymphangiogenesis. Tumor cells directly bind to the VEGFC promoter E-box region via c-Myc and improve VEGFR3 phosphorylation to augment lymphatic endothelial cell tube formation, hence amplifying lymph node metastasis in pancreatic NETs [96]. Additionally, pseudo-hemorrhage was observed with characteristics demonstrating how abundant vessel endothelial cells were divorced from vessel dilation to create blood-filled caverns with no endothelial cell boundary in insulinoma, and it was encircled by tumor cells with increased E-cadherin and β-catenin expression [97], implying that it was connected to the epithelial–mesenchymal transition process of tumor cells. The tumor cells continued to destroy the body’s organs and tissues through blood, lymph, and other channels. Consequently, treatments targeting regulatory molecules for stifling the formation of these channels is likely to significantly ameliorate patient outcomes.

## 6. Microbial Community

The microbiota plays a pivotal role in supporting energy balance and health maintenance. In fact, 75% of patients with intestinal NENs showed bacterial infiltration within the tumor tissue, while 90% of pancreatic NEN specimens showed quantifiable microbial tissue infiltration [98]. This shows the prevalence of microbial invasion in NENs. Hu et al. profiled fecal samples for rectal NETs [99]. The results of that study are consistent with the general trend of the significant depletion of microbial species diversity in NETs. Bacteria, including *Haemophilus parainfluenzae*, *Veillonella* unclassified, and *Streptococcus salivarius,* were significantly enriched in the healthy group, while species such as *Erysipelotrichaceae bacterium_6_1_45*, *Varibaculum cambriense*, and *Methanobrevibacter smithii* were abundant in the rectal NET group [99]. For the functional characterization of the microbiome, glycerophospholipid metabolism as a key tumor-specific pathway is distinctively aberrant in rectal NET patients and is positively correlated with *Methanobrevibacter smithii*, which is mainly due to changes in the proportion of microbial species [99]. The study implies that metagenome-based physiological dysfunction in the intestinal microenvironment drives the disease-progression state of individuals with rectal NETs. For viruses, two kinds of GEP-NECs are etiologically related to viruses: MCPyV acts on gastric NECs, and HPV acts on colorectal NECs [100]. However, reports of fungal involvement in tumors remain to be explored.

Oncolytic viruses are new therapeutic agents for cancer. These viruses occur naturally or have been modified to selectively infect targeted tumor cells, producing large numbers of viral progeny that result in the accelerated lysis of tumor cells. The important mechanism of oncolytic virotherapy is thought to be the secondary antitumor immune response to changes in the TME induced by tumor cell lysis products [101]. Oncolytic adenovirus Ad5 (CgA-E1A-miR122) selectively replicates in NET cells and does not destroy normal liver cells. Moreover, by integrating the binding motif of the Phe-Trp-Lys-Thr ring into the Ad5 fiber button, the Ad5fk^FWKT^(CgA-E1A-miR122) is modified to target somatostatin receptor-positive NET cells, further improving the transduction and killing effect [102]. In order to avoid the problem of excessive fiber production and secretion after virus infection, this team developed a hexon Tat-protein transduction domain-modified oncolytic Ad5 virus that is able to display higher NET tumor inhibition [103]. These preclinical studies provide the basis for the ongoing phase I/IIa clinical trial of this AdVince oncolytic virus (NCT02749331). Another oncolytic virus, AdSur-SYE, was designed and showed high gene transduction efficiency against pancreatic NETs, reducing the number of subcutaneous tumors in mouse models [104]. Talimogene laherparepvec is a first-generation, state-of-the-art HSV-1-based oncolytic virus that does not integrate its viral DNA into the host genome and that shows high oncolytic efficacy at low concentrations when applied to NETs and NECs [105]. GLV-1 h68 relies on vaccinia viruses that also do not disturb the host genome and have a good oncolytic effect against NETs and NECs [106].

## 7. Other Cells and Molecules in TME

Other cells and molecules, such as fibroblasts and the extracellular matrix (Figure 1), play an important role in tumor growth, invasion, and therapy response [107]. Neural invasion is also frequent in high-grade tumors and is highly associated with encroachment in vessels, organs, and lymph nodes [108]. Tumor-infiltrating platelets are independent poor predictors of overall and recurrence-free survival in pancreatic NETs [109]. Immune cytokines and chemokines also have effects. Despite CCL5 being considered an immunosuppressive molecule, it is associated with frequent CD8^+^ T cell infiltration and extends survival in colorectal NECs [34]. CXCR4 gradually escalates in GEP-NETs, with 0% in G1 and 80% in G3 diseases, meaning that it could be selected as a potential therapeutic and positron emission tomography/computed tomography imaging target [110]. Interferon-α and interferon-β are promising adjuvant therapies for GEP-NENs but have unfavorable toxicity [4]. However, there are currently still relatively few relevant studies on other cells and molecules, and their roles have not been fully revealed, and further studies are required to elucidate them.

Due to inflammation and fibrosis, activated fibroblasts are recruited to the tumor site. The single-cell RNA sequencing of pancreatic NET (G2) from the same patient revealed that fibroblasts could be roughly divided into inflammatory, antigen-presenting, and myofibroblastic cancer-associated fibroblasts, some of which include inflammatory subtypes that can secrete cellular senescence factors [18]. It also found that the TME of primary tumors contained more heterogeneous fibroblast clusters than those of liver metastases; however, there were also fibroblasts specific to liver metastases [18]. The crosstalk between NEN cells and fibroblasts induces excessive fibrosis, and the most common example of fibrosis is mesenteric fibrosis. Mesenteric fibrosis may occur in some patients but more often occurs in NETs with mesenteric vessel encasement, hepatic metastases, larger hepatic tumor burden, and functionality [111]. They partially occur due to serotonin, growth factors, and other cytokines released from tumor cells via the activation of the ingenuity-associated MAPK and mTOR pathways [112] and then react on fibroblasts [113,114]. Nevertheless, preventive surgery or mesenteric lump metastasectomy did not contribute to overall survival [115,116]. One possible reason might be that both fibroblasts and collagen are double-sided and can promote both tumor cell progression and inhibit tumor cell proliferation [117]. Thus, the specific treatment strategy or specifically targeted treatment for mesenteric fibrosis still needs to be explored and discovered.

## 8. TME-Associated Combination Treatment

The effective rate of monotherapy or a single class of drugs is relatively low, but the combination of immunotherapy, chemotherapy, radiotherapy, targeted therapy, and other therapies has initially achieved good therapeutic effects in many tumors. Combination therapy has the advantages of a synergistic treatment mechanism, a reduced drug dose, and enhanced therapeutic effects, demonstrating a promising tumor treatment method.

Antiangiogenic agents strengthen antitumor immunity by suppressing various angiogenesis dysplastic immune processes [118]. Therefore, when combined with immunotherapy, antiangiogenic drugs may produce better clinical outcomes, and there are a number of clinical trials that are currently in progress (Table 3). It should be noted that the VEGFR family has different connections with immune checkpoints because though VEGFR1 expression is not associated with PD-1 and PD-L1 expression, VEGFR2 and VEGFR3 are related to PD-1 and PD-L1, respectively [87].

As more drugs or multimodality therapies for TME are developed, combinations of these drugs or supplements with existing first-line therapies could represent a better feasible option. A tyrosine kinase inhibitor vorolanib showed safe toxicity and antitumor efficacy when used in combination with everolimus [119]. Bevacizumab and temsirolimus confirmed good tolerability and an ORR of 41% in pancreatic NETs [120]. Moreover, one study on patients with NECs found that checkpoint inhibitor monotherapy (pembrolizumab, nivolumab, or atezolizumab) had an ORR worse than 0%, while dual nivolumab and ipilimumab treatment showed a 13% ORR, and combined therapy: platinum-based therapy with checkpoint inhibitors, demonstrated a 36% ORR [121]. Combined therapy reached a median progression-free survival rate of 4.2 months compared to the 2.1 months achieved by monotherapy [121]. However, drug combinations still need to focus on the occurrence of adverse reactions. Sunitinib and evofosfamide had 88.2% systemic toxicity, failing in phase II clinical trials for G1/2 in metastatic pancreatic NETs [122]. During combined use, attention should be paid to the differences in pharmacokinetic properties and the in vivo distribution of different drugs, insufficient tumor accumulation, uncertain drug interactions in tumor tissues, and serious side effects. There are also many ongoing clinical trials for combinations of antiangiogenic drugs or immune checkpoint inhibitors combined with existing therapeutic regimens (Table 4). Another noteworthy combination regimen is oncolytic virus immunotherapy. Oncolytic vaccinia virus mpJX-594 combined with the PD-1 antibody can induce an extensive influx of cytotoxic T cells into tumor tissues, inhibit tumor proliferation, and subside liver metastasis, thereby increasing survival time in pancreatic NET mice models [123].

## 9. TME-Associated Clinical Application Prospects

TME-associated treatment can make the tumorous living environment worse, making it not conducive to tumor occurrence and development. On the other hand, it could reverse the antitumor immune response to exerting the effect of killing tumor cells.

Few studies on GEP-NENs have concentrated specifically on the role of B cells, dendritic cells, collagen, and so on, although they have an important influence on tumor occurrence and development, which are extensively studied in many tumors [124]. Targeting macrophages has been investigated in clinical trials for other tumor types [15]. Modulating Treg cells as an alternative treatment is being investigated in many tumor types [125]. In NENs, immune responses are protumorigenic and antitumorigenic [126], and immunomodulatory factors (CD45, IL2RB1, CD53, CD86, RUNX3, CIITA, and IL10) are also key hallmarks of aggressive GEP-NENs [127]. More cell- and molecule-associated studies should be considered when conducting preclinical studies and clinical trials.

Additionally, other strategies are incompletely developed in GEP-NENs. Adoptive cell therapies such as chimeric antigen receptor (CAR)-T and TCR-T immunotherapy, which rely on reprogramming the patient’s own extracted T cells to specifically kill tumor cells, are becoming common treatments for many tumors, and cytokines such as IL-2 can be injected to activate immune effector cells [128]. All of these approaches can also be attempted in NENs. CDH17-dependent CAR T cells destroy pancreatic NET cells but do not violate normal intestinal epithelial cells, suggesting that CAR T therapy may be promising immunotherapy for NENs [129]. Novel autologous DC vaccines and SVN53-67/M57-KLH peptide vaccines (SurVaxM) in emulsions are currently being used to explore safety and efficacy in clinical trials (NCT04166006, NCT03879694).

Nano-based drug delivery systems include liposomes, polymer micelles, dendrimers, metallic and inorganic nanoparticles, nanogels, and biomimetic nanoparticles, and they can improve the pharmacokinetic behavior of drugs in vivo, increase drug stability, and achieve targeted drug delivery and controlled drug release [130]. These new technologies and new pharmaceutical materials are expected to be important means of treating diseases.

Moreover, more new technologies involving single-cell sequencing and spatial transcriptome analysis should be utilized to further reveal the state of the TME [131]. Multiple omics methods are used to reveal and analyze pancreatic NET specimens, with four subgroups having been identified: proliferation, PDX1-high, alpha cell-like, and stromal/mesenchymal subsets, and the stromal/mesenchymal subsets are rich in stromal and immune cells and are related to the molecular characterization of YAP1/WWTR1 (TAZ) activation, suggesting the role of the Hippo signaling pathways in this process [132]. Based on these new technologies, related targeted therapies are promising. At present, there is a lack of specific biomarkers to predict treatment efficacy in GEP-NENs, and the changes in the TME can be regarded as a treatment indication or imaging option.

The diagnosis of NENs depends on determining morphologies and neuroendocrine cell differentiation. In 2019 and 2022, the World Health Organization further defined NEN classification into NETs and NECs, which are different from the 2010 classification criteria that defined all high-grade NENs as NECs [133]. However, distinguishing high-grade (G3) NETs from NECs remains challenging through the use of morphology and a proliferative index. Therefore, the evaluation of other characteristics and ancillary studies are necessary to discriminate between G3 NETs and NECs. However, little literature exists on the differences between the two from the perspective of the TME. Notably, one study points out that some subsets of NETs are more similar to NECs at the molecular level than to other NETs, indicating that there is still some subset overlap between NETs and NECs, which may be part of the reason why it is difficult to distinguish between the two [132]. More refined large sample studies are needed to clarify the boundary between G3 NETs and NECs.

## 10. Conclusions

NENs can be subtyped based on their gene-expression profile and TME phenotype, and the accurate pathological interpretation of a specific tumor is important for treatment and prognosis. We also need to determine the timing of NEN evolution and the role of genetic heterogeneity within the TME. Regulating the TME can represent innovative therapeutic strategies for NENs to prevent tumors from immune escape and to support antitumoral effects. According to the characteristics of tumor heterogeneity, it is necessary to adopt a personalized and precise therapeutic regimen to balance all aspects of the tumor to prolong patient survival and reduce the occurrence of adverse events.

## Figures and Tables

**Table 1:** Ongoing or activated clinical trials of immune checkpoint-associated immunotherapy in patients with gastroenteropancreatic NENs.

NCT Number	Conditions	Drugs	Targets	Phases	Primary Endpoints
NCT03517488	Progressive NECs	XmAb^®^20717	PD-1/CTLA-4 bispecific antibody	1	Safety, tolerability
NCT03012620	Progressive NETs	Pembrolizumab	PD-1	2	Objective response rate
NCT03352934	Progressive NECs	Avelumab	PD-L1	2	Disease control rate
NCT03278379	Progressive NETs with G2/3	Avelumab	PD-L1	2	Overall response rate
NCT03591731	Progressive NECs	Nivolumab +/− ipilimumab	PD-1, CTLA-4	2	Objective response rate
NCT03420521	Progressive well-differentiated nonfunctional NETs	Nivolumab, ipilimumab	PD-1, CTLA-4	2	Objective response rate
NCT03095274	Progressive NENs	Durvalumab, tremelimumab	PD-L1, CTLA-4	2	Clinical benefit rate

**Table 2:** Ongoing or activated phase II and III clinical trials of multiple-receptor tyrosine kinase inhibitor monotherapy in patients with gastroenteropancreatic neuroendocrine neoplasms.

NCT Number	Conditions	Drugs	Phases	Primary Endpoints
NCT02549937	Progressive NETs	Surufatinib	1, 2	Dose-limiting toxicity incidence, progression-free survival
NCT04579679	Progressive NETs	Surufatinib	2	Disease control rate
NCT03457844	Progressive NETs with G3 and NECs	Anlotinib	2	Progression-free survival
NCT04524208	Locally unresectable or metastatic NENs with Ki67 of 20–60%	Cabozantinib	2	Disease control rate
NCT04412629	Progressive NETs with G3 and NECs	Cabozantinib	2	Objective response rate
NCT01466036	Locally unresectable or metastatic, well-differentiated, pancreatic NETs	Cabozantinib	2	objective response rate
NCT03375320	Locally unresectable or metastatic NETs with G1/2	Cabozantinib S-malate	3	Progression-free survival

**Table 3:** Ongoing or activated clinical trials of antiangiogenic drugs combined with immunotherapy in patients with gastroenteropancreatic neuroendocrine neoplasms.

NCT Number	Conditions	Drugs	Phases	Primary Endpoints
NCT05015621	Progressive NECs	Surufatnib, toripalimab	3	Overall survival
NCT04207463	Late NETs with G1/2	Anlotinib, penpulimab,	2	Overall response rate
NCT04400474	Progressive NETs with G3 and NECs	Cabozantinib, atezolizumab	2	Objective response rate
NCT04197310	Locally unresectable or metastatic well-differentiated, non-pancreatic NETs	Cabozantinib, nivolumab	2	Objective response rate
NCT04079712	Progressive poorly-differentiated NETs and NECs	Cabozantinib s-malate, nivolumab, ipilimumab	2	Overall response rate
NCT03290079	Progressive well-differentiated NETs of small intestinal and colorectal origin	Lenvatinib, pembrolizumab	2	Objective response rate
NCT03074513	Progressive NETs with G1/2	Bevacizumab, atezolizumab	2	Objective response rate

**Table 4:** Ongoing or activated clinical trials of antiangiogenic drugs or immune checkpoint inhibitors combined with existing therapeutic regimens in patients with gastroenteropancreatic neuroendocrine neoplasms.

NCT Number	Conditions	Drugs	Combinations	Phases	Primary Endpoints
NCT05048901	Progressive NETs	Cabozantinib	Lanreotide	1,2	Maximal tolerated dose, progression-free survival
NCT04427787	Locally unresectable or metastatic, well-differentiated NETs	Cabozantinib	Lanreotide	2	Objective response rate, safety
NCT04893785	Progressive NETs and large cells NECs with Ki67< 55%	Cabozantinib	Temozolomide	2	Overall response rate
NCT02230176	Progressive pancreatic NETs	Sunitinib	177Lu-dota0-Tyr3-octreotate	2	Progression-free survival
NCT03950609	Locally unresectable or metastatic NETs with G1/2	Lenvatinib	Everolimus	2	Radiographic response rate, objective response rate
NCT02820857	Progressive NECs	Bevacizumab	FOLFIRI	2	proportion of patients alive 6 months after treatment
NCT04705519	Progressive NECs	Bevacizumab	Nab-paclitaxel	2	Overall Survival
NCT01782443	Progressive NETs with G1/2	Ziv-aflibercept	Octreotide LAR	2	Progression-free survival
NCT04525638	Locally unresectable, recurrent or metastatic NETs with G3 and NECs	Nivolumab	177Lu-dotatate	2	Overall response rate
NCT03980925	Locally unresectable or metastatic, NETs with G3 and NECs	Nivolumab	Carboplatin, etoposide	2	Overall survival
NCT03728361	Progressive NECs	Nivolumab	Temozolomide	2	Objective response rate
NCT05058651	Small cell NECs	Atezolizumab	Platinum drug (cisplatin or carboplatin) and etoposide	2, 3	Overall survival
NCT03457948	Progressive NETs	Pembrolizumab	177Lu-dota0-Tyr3-octreotate, or arterial embolization, or yttrium-90 microsphere radioembolization	2	Overall response rate
NCT03136055	Progressive NECs	Pembrolizumab	Irinotecan, paclitaxel	2	Overall response rate
NCT03043664	Progressive NETs with G1/2	Pembrolizumab	Lanreotide	1,2	Overall response rate
NCT02489903	Progressive NETs with G3 and NECs	RRx-001	Platinum based doublet regimen	2	Overall survival

Drugs refer to antiangiogenic drugs or immune checkpoint inhibitors. Combinations refer to existing therapeutic regimens excluding antiangiogenic drugs or immune checkpoint inhibitors.

## Data Availability

Not applicable.

## References

[1] Dasari A., Shen C., Halperin D., Zhao B., Zhou S., Xu Y., Shih T., Yao J.C. (2017). Trends in the Incidence, Prevalence, and Survival Outcomes in Patients with Neuroendocrine Tumors in the United States. JAMA Oncol..

[2] Cives M., Strosberg J.R. (2018). Gastroenteropancreatic Neuroendocrine Tumors. CA Cancer J. Clin..

[3] Mafficini A., Scarpa A. (2019). Genetics and Epigenetics of Gastroenteropancreatic Neuroendocrine Neoplasms. Endocr. Rev..

[4] Pavel M., Oberg K., Falconi M., Krenning E.P., Sundin A., Perren A., Berruti A. (2020). Gastroenteropancreatic neuroendocrine neoplasms: ESMO Clinical Practice Guidelines for diagnosis, treatment and follow-up. Ann. Oncol..

[5] Hanahan D., Weinberg R.A. (2011). Hallmarks of cancer: The next generation. Cell.

[6] Xiao Y., Yu D. (2021). Tumor microenvironment as a therapeutic target in cancer. Pharmacol. Ther..

[7] Yang L., Lin P.C. (2017). Mechanisms that drive inflammatory tumor microenvironment, tumor heterogeneity, and metastatic progression. Semin. Cancer Biol..

[8] Topalian S.L., Taube J.M., Pardoll D.M. (2020). Neoadjuvant checkpoint blockade for cancer immunotherapy. Science.

[9] Heinhuis K.M., Ros W., Kok M., Steeghs N., Beijnen J.H., Schellens J.H.M. (2019). Enhancing antitumor response by combining immune checkpoint inhibitors with chemotherapy in solid tumors. Ann. Oncol..

[10] Xu J., Shen L., Zhou Z., Li J., Bai C., Chi Y., Li Z., Xu N., Li E., Liu T. (2020). Surufatinib in advanced extrapancreatic neuroendocrine tumours (SANET-ep): A randomised, double-blind, placebo-controlled, phase 3 study. Lancet Oncol..

[11] Raymond E., Dahan L., Raoul J.L., Bang Y.J., Borbath I., Lombard-Bohas C., Valle J., Metrakos P., Smith D., Vinik A. (2011). Sunitinib malate for the treatment of pancreatic neuroendocrine tumors. N. Engl. J. Med..

[12] Couvelard A., Cros J. (2022). An update on the development of concepts, diagnostic criteria, and challenging issues for neuroendocrine neoplasms across different digestive organs. Virchows Arch..

[13] Rothlin C.V., Ghosh S. (2020). Lifting the innate immune barriers to antitumor immunity. J. Immunother. Cancer.

[14] Centonze G., Lagano V., Sabella G., Mangogna A., Garzone G., Filugelli M., Belmonte B., Cattaneo L., Crisafulli V., Pellegrinelli A. (2021). Myeloid and T-Cell Microenvironment Immune Features Identify Two Prognostic Sub-Groups in High-Grade Gastroenteropancreatic Neuroendocrine Neoplasms. J. Clin. Med..

[15] DeNardo D.G., Ruffell B. (2019). Macrophages as regulators of tumour immunity and immunotherapy. Nat. Rev. Immunol..

[16] Ferrata M., Schad A., Zimmer S., Musholt T.J., Bahr K., Kuenzel J., Becker S., Springer E., Roth W., Weber M.M. (2019). PD-L1 Expression and Immune Cell Infiltration in Gastroenteropancreatic (GEP) and Non-GEP Neuroendocrine Neoplasms with High Proliferative Activity. Front. Oncol..

[17] Krug S., Abbassi R., Griesmann H., Sipos B., Wiese D., Rexin P., Blank A., Perren A., Haybaeck J., Huttelmaier S. (2018). Therapeutic targeting of tumor-associated macrophages in pancreatic neuroendocrine tumors. Int. J. Cancer.

[18] Zhou Y., Liu S., Liu C., Yang J., Lin Q., Zheng S., Chen C., Zhou Q., Chen R. (2021). Single-cell RNA sequencing reveals spatiotemporal heterogeneity and malignant progression in pancreatic neuroendocrine tumor. Int. J. Biol. Sci..

[19] Harimoto N., Hoshino K., Muranushi R., Hagiwara K., Yamanaka T., Ishii N., Tsukagoshi M., Igarashi T., Tanaka H., Watanabe A. (2019). Prognostic significance of neutrophil-lymphocyte ratio in resectable pancreatic neuroendocrine tumors with special reference to tumor-associated macrophages. Pancreatology.

[20] Wei I.H., Harmon C.M., Arcerito M., Cheng D.F., Minter R.M., Simeone D.M. (2014). Tumor-associated macrophages are a useful biomarker to predict recurrence after surgical resection of nonfunctional pancreatic neuroendocrine tumors. Ann. Surg..

[21] Cai L., Michelakos T., Deshpande V., Arora K.S., Yamada T., Ting D.T., Taylor M.S., Castillo C.F., Warshaw A.L., Lillemoe K.D. (2019). Role of Tumor-Associated Macrophages in the Clinical Course of Pancreatic Neuroendocrine Tumors (PanNETs). Clin. Cancer Res..

[22] Xu S.S., Li H., Li T.J., Li S., Xia H.Y., Long J., Wu C.T., Wang W.Q., Zhang W.H., Gao H.L. (2021). Neutrophil Extracellular Traps and Macrophage Extracellular Traps Predict Postoperative Recurrence in Resectable Nonfunctional Pancreatic Neuroendocrine Tumors. Front. Immunol..

[23] Schiavo Lena M., Partelli S., Castelli P., Andreasi V., Smart C.E., Pisa E., Bartolomei M., Bertani E., Zamboni G., Falconi M. (2020). Histopathological and Immunophenotypic Changes of Pancreatic Neuroendocrine Tumors after Neoadjuvant Peptide Receptor Radionuclide Therapy (PRRT). Endocr. Pathol..

[24] Cassetta L., Pollard J.W. (2018). Targeting macrophages: Therapeutic approaches in cancer. Nat. Rev. Drug Discov..

[25] Akkari L., Gocheva V., Kester J.C., Hunter K.E., Quick M.L., Sevenich L., Wang H.W., Peters C., Tang L.H., Klimstra D.S. (2014). Distinct functions of macrophage-derived and cancer cell-derived cathepsin Z combine to promote tumor malignancy via interactions with the extracellular matrix. Genes Dev..

[26] Harney A.S., Karagiannis G.S., Pignatelli J., Smith B.D., Kadioglu E., Wise S.C., Hood M.M., Kaufman M.D., Leary C.B., Lu W.P. (2017). The Selective Tie_2_ Inhibitor Rebastinib Blocks Recruitment and Function of Tie_2_(Hi) Macrophages in Breast Cancer and Pancreatic Neuroendocrine Tumors. Mol. Cancer Ther..

[27] Pyonteck S.M., Gadea B.B., Wang H.W., Gocheva V., Hunter K.E., Tang L.H., Joyce J.A. (2012). Deficiency of the macrophage growth factor CSF-1 disrupts pancreatic neuroendocrine tumor development. Oncogene.

[28] Coffelt S.B., Wellenstein M.D., de Visser K.E. (2016). Neutrophils in cancer: Neutral no more. Nat. Rev. Cancer.

[29] Reid M.D., Basturk O., Thirabanjasak D., Hruban R.H., Klimstra D.S., Bagci P., Altinel D., Adsay V. (2011). Tumor-infiltrating neutrophils in pancreatic neoplasia. Mod. Pathol..

[30] Zhang W.H., Wang W.Q., Gao H.L., Xu S.S., Li S., Li T.J., Han X., Xu H.X., Li H., Jiang W. (2020). Tumor-Infiltrating Neutrophils Predict Poor Survival of Non-Functional Pancreatic Neuroendocrine Tumor. J. Clin. Endocrinol. Metab..

[31] Debien V., Davidson G., Baltzinger P., Kurtz J.E., Severac F., Imperiale A., Pessaux P., Addeo P., Bachellier P., Su X. (2021). Involvement of Neutrophils in Metastatic Evolution of Pancreatic Neuroendocrine Tumors. Cancers.

[32] Soncini M., Corna G., Moresco M., Coltella N., Restuccia U., Maggioni D., Raccosta L., Lin C.Y., Invernizzi F., Crocchiolo R. (2016). 24-Hydroxycholesterol participates in pancreatic neuroendocrine tumor development. Proc. Natl. Acad. Sci. USA.

[33] Liu M., Zhang Y., Chen L., Lin Y., He Q., Zeng Y., Chen M., Chen J. (2021). Myeloid-derived suppressor cells in gastroenteropancreatic neuroendocrine neoplasms. Endocrine.

[34] Chen D., Bao X., Zhang R., Ding Y., Zhang M., Li B., Zhang H., Li X., Tong Z., Liu L. (2021). Depiction of the genomic and genetic landscape identifies CCL5 as a protective factor in colorectal neuroendocrine carcinoma. Br. J. Cancer.

[35] Kosaloglu Z., Zornig I., Halama N., Kaiser I., Buchhalter I., Grabe N., Eils R., Schlesner M., Califano A., Jager D. (2016). Identification of immunotherapeutic targets by genomic profiling of rectal NET metastases. Oncoimmunology.

[36] Soucek L., Buggy J.J., Kortlever R., Adimoolam S., Monclus H.A., Allende M.T., Swigart L.B., Evan G.I. (2011). Modeling pharmacological inhibition of mast cell degranulation as a therapy for insulinoma. Neoplasia.

[37] Al-Toubah T., Schell M.J., Cives M., Zhou J.M., Soares H.P., Strosberg J.R. (2020). A Phase II Study of Ibrutinib in Advanced Neuroendocrine Neoplasms. Neuroendocrinology.

[38] Mo S., Zong L., Chen X., Chang X., Lu Z., Yu S., Chen J. (2021). High Mast Cell Density Predicts a Favorable Prognosis in Patients with Pancreatic Neuroendocrine Neoplasms. Neuroendocrinology.

[39] Szabo P.A., Levitin H.M., Miron M., Snyder M.E., Senda T., Yuan J., Cheng Y.L., Bush E.C., Dogra P., Thapa P. (2019). Single-cell transcriptomics of human T cells reveals tissue and activation signatures in health and disease. Nat. Commun..

[40] Borst J., Ahrends T., Babala N., Melief C.J.M., Kastenmuller W. (2018). CD4(+) T cell help in cancer immunology and immunotherapy. Nat. Rev. Immunol..

[41] Bosch F., Bruwer K., Altendorf-Hofmann A., Auernhammer C.J., Spitzweg C., Westphalen C.B., Boeck S., Schubert-Fritschle G., Werner J., Heinemann V. (2019). Immune checkpoint markers in gastroenteropancreatic neuroendocrine neoplasia. Endocr. Relat. Cancer.

[42] Baretti M., Zhu Q., Zahurak M., Bhaijee F., Xu H., Engle E.L., Kotte A., Pawlik T.M., Anders R.A., De Jesus-Acosta A. (2021). Prognostic Implications of the Immune Tumor Microenvironment in Patients with Pancreatic and Gastrointestinal Neuroendocrine Tumors. Pancreas.

[43] Sato S., Tsuchikawa T., Nakamura T., Sato N., Tamoto E., Okamura K., Shichinohe T., Hirano S. (2014). Impact of the tumor microenvironment in predicting postoperative hepatic recurrence of pancreatic neuroendocrine tumors. Oncol. Rep..

[44] Da Silva A., Bowden M., Zhang S., Masugi Y., Thorner A.R., Herbert Z.T., Zhou C.W., Brais L., Chan J.A., Hodi F.S. (2018). Characterization of the Neuroendocrine Tumor Immune Microenvironment. Pancreas.

[45] Busse A., Mochmann L.H., Spenke C., Arsenic R., Briest F., Johrens K., Lammert H., Sipos B., Kuhl A.A., Wirtz R. (2020). Immunoprofiling in Neuroendocrine Neoplasms Unveil Immunosuppressive Microenvironment. Cancers.

[46] De Reuver P.R., Mehta S., Gill P., Andrici J., D’Urso L., Clarkson A., Mittal A., Hugh T.J., Samra J.S., Gill A.J. (2016). Immunoregulatory Forkhead Box Protein p3-Positive Lymphocytes Are Associated with Overall Survival in Patients with Pancreatic Neuroendocrine Tumors. J. Am. Coll. Surg..

[47] Sciammarella C., Luce A., Riccardi F., Mocerino C., Modica R., Berretta M., Misso G., Cossu A.M., Colao A., Vitale G. (2020). Lanreotide Induces Cytokine Modulation in Intestinal Neuroendocrine Tumors and Overcomes Resistance to Everolimus. Front. Oncol..

[48] Tanno L., Naheed S., Dunbar J., Tod J., Lopez M.A., Taylor J., Machado M., Green B., Ashton-Key M., Chee S.J. (2021). Analysis of Immune Landscape in Pancreatic and Ileal Neuroendocrine Tumours Demonstrates an Immune Cold Tumour Microenvironment. Neuroendocrinology.

[49] Zhang W.H., Wang W.Q., Han X., Gao H.L., Xu S.S., Li S., Li T.J., Xu H.X., Li H., Ye L.Y. (2020). Infiltrating pattern and prognostic value of tertiary lymphoid structures in resected non-functional pancreatic neuroendocrine tumors. J. Immunother. Cancer.

[50] Sautes-Fridman C., Lawand M., Giraldo N.A., Kaplon H., Germain C., Fridman W.H., Dieu-Nosjean M.C. (2016). Tertiary Lymphoid Structures in Cancers: Prognostic Value, Regulation, and Manipulation for Therapeutic Intervention. Front. Immunol..

[51] Engelhard V.H., Rodriguez A.B., Mauldin I.S., Woods A.N., Peske J.D., Slingluff C.L. (2018). Immune Cell Infiltration and Tertiary Lymphoid Structures as Determinants of Antitumor Immunity. J. Immunol..

[52] Rosery V., Reis H., Savvatakis K., Kowall B., Stuschke M., Paul A., Dechene A., Yang J., Zhao B., Borgers A. (2021). Antitumor immune response is associated with favorable survival in GEP-NEN G3. Endocr. Relat. Cancer.

[53] Roberts J.A., Gonzalez R.S., Das S., Berlin J., Shi C. (2017). Expression of PD-1 and PD-L1 in poorly differentiated neuroendocrine carcinomas of the digestive system: A potential target for anti-PD-1/PD-L1 therapy. Hum. Pathol..

[54] Puccini A., Poorman K., Salem M.E., Soldato D., Seeber A., Goldberg R.M., Shields A.F., Xiu J., Battaglin F., Berger M.D. (2020). Comprehensive Genomic Profiling of Gastroenteropancreatic Neuroendocrine Neoplasms (GEP-NENs). Clin. Cancer Res..

[55] Milione M., Miceli R., Barretta F., Pellegrinelli A., Spaggiari P., Tagliabue G., Centonze G., Paolino C., Mangogna A., Kankava K. (2019). Microenvironment and tumor inflammatory features improve prognostic prediction in gastro-entero-pancreatic neuroendocrine neoplasms. J. Pathol. Clin. Res..

[56] Cavalcanti E., Armentano R., Valentini A.M., Chieppa M., Caruso M.L. (2017). Role of PD-L1 expression as a biomarker for GEP neuroendocrine neoplasm grading. Cell Death Dis..

[57] Cives M., Strosberg J., Al Diffalha S., Coppola D. (2019). Analysis of the immune landscape of small bowel neuroendocrine tumors. Endocr. Relat. Cancer.

[58] Yang M.W., Fu X.L., Jiang Y.S., Chen X.J., Tao L.Y., Yang J.Y., Huo Y.M., Liu W., Zhang J.F., Liu P.F. (2019). Clinical significance of programmed death 1/programmed death ligand 1 pathway in gastric neuroendocrine carcinomas. World J. Gastroenterol..

[59] Taboada R., Claro L., Felismino T., de Jesus V.H., Barros M., Riechelmann R.P. (2022). Clinicopathological and molecular profile of grade 3 gastroenteropancreatic neuroendocrine neoplasms. J. Neuroendocrinol..

[60] Fraune C., Simon R., Hube-Magg C., Makrypidi-Fraune G., Kluth M., Buscheck F., Amin T., Viol F., Fehrle W., Dum D. (2020). Homogeneous MMR Deficiency Throughout the Entire Tumor Mass Occurs in a Subset of Colorectal Neuroendocrine Carcinomas. Endocr. Pathol..

[61] Xing J., Ying H., Li J., Gao Y., Sun Z., Li J., Bai C., Cheng Y., Wu H. (2020). Immune Checkpoint Markers in Neuroendocrine Carcinoma of the Digestive System. Front. Oncol..

[62] Salem M.E., Puccini A., Grothey A., Raghavan D., Goldberg R.M., Xiu J., Korn W.M., Weinberg B.A., Hwang J.J., Shields A.F. (2018). Landscape of Tumor Mutation Load, Mismatch Repair Deficiency, and PD-L1 Expression in a Large Patient Cohort of Gastrointestinal Cancers. Mol. Cancer Res..

[63] Pinato D.J., Vallipuram A., Evans J.S., Wong C., Zhang H., Brown M., Dina R.E., Trivedi P., Akarca A.U., Marafioti T. (2021). Programmed Cell Death Ligand Expression Drives Immune Tolerogenesis across the Diverse Subtypes of Neuroendocrine Tumours. Neuroendocrinology.

[64] Lu M., Zhang P., Zhang Y., Li Z., Gong J., Li J., Li J., Li Y., Zhang X., Lu Z. (2020). Efficacy, Safety, and Biomarkers of Toripalimab in Patients with Recurrent or Metastatic Neuroendocrine Neoplasms: A Multiple-Center Phase Ib Trial. Clin. Cancer Res..

[65] Vijayvergia N., Dasari A., Deng M., Litwin S., Al-Toubah T., Alpaugh R.K., Dotan E., Hall M.J., Ross N.M., Runyen M.M. (2020). Pembrolizumab monotherapy in patients with previously treated metastatic high-grade neuroendocrine neoplasms: Joint analysis of two prospective, non-randomised trials. Br. J. Cancer.

[66] Strosberg J., Mizuno N., Doi T., Grande E., Delord J.P., Shapira-Frommer R., Bergsland E., Shah M., Fakih M., Takahashi S. (2020). Efficacy and Safety of Pembrolizumab in Previously Treated Advanced Neuroendocrine Tumors: Results from the Phase II KEYNOTE-158 Study. Clin. Cancer Res..

[67] MacFarlane A.W.T., Yeung H.M., Alpaugh R.K., Dulaimi E., Engstrom P.F., Dasari A., Campbell K.S., Vijayvergia N. (2021). Impacts of pembrolizumab therapy on immune phenotype in patients with high-grade neuroendocrine neoplasms. Cancer Immunol. Immunother..

[68] Yap T.A., Parkes E.E., Peng W., Moyers J.T., Curran M.A., Tawbi H.A. (2021). Development of Immunotherapy Combination Strategies in Cancer. Cancer Discov..

[69] Patel S.P., Othus M., Chae Y.K., Giles F.J., Hansel D.E., Singh P.P., Fontaine A., Shah M.H., Kasi A., Baghdadi T.A. (2020). A Phase II Basket Trial of Dual Anti-CTLA-4 and Anti-PD-1 Blockade in Rare Tumors (DART SWOG 1609) in Patients with Nonpancreatic Neuroendocrine Tumors. Clin. Cancer Res..

[70] Klein O., Kee D., Markman B., Michael M., Underhill C., Carlino M.S., Jackett L., Lum C., Scott C., Nagrial A. (2020). Immunotherapy of Ipilimumab and Nivolumab in Patients with Advanced Neuroendocrine Tumors: A Subgroup Analysis of the CA209-538 Clinical Trial for Rare Cancers. Clin. Cancer Res..

[71] Patel S.P., Mayerson E., Chae Y.K., Strosberg J., Wang J., Konda B., Hayward J., McLeod C.M., Chen H.X., Sharon E. (2021). A phase II basket trial of Dual Anti-CTLA-4 and Anti-PD-1 Blockade in Rare Tumors (DART) SWOG S1609: High-grade neuroendocrine neoplasm cohort. Cancer.

[72] Al-Toubah T., Halfdanarson T., Gile J., Morse B., Sommerer K., Strosberg J. (2022). Efficacy of ipilimumab and nivolumab in patients with high-grade neuroendocrine neoplasms. ESMO Open.

[73] Yao J.C., Strosberg J., Fazio N., Pavel M.E., Bergsland E., Ruszniewski P., Halperin D.M., Li D., Tafuto S., Raj N. (2021). Spartalizumab in metastatic, well/poorly-differentiated neuroendocrine neoplasms. Endocr. Relat. Cancer.

[74] Marin-Acevedo J.A., Kimbrough E.O., Lou Y. (2021). Next generation of immune checkpoint inhibitors and beyond. J. Hematol. Oncol..

[75] Lee J.B., Ha S.J., Kim H.R. (2021). Clinical Insights into Novel Immune Checkpoint Inhibitors. Front. Pharmacol..

[76] Yuan Z., Gardiner J.C., Maggi E.C., Huang S., Adem A., Bagdasarov S., Li G., Lee S., Slegowski D., Exarchakis A. (2021). B7 immune-checkpoints as targets for the treatment of neuroendocrine tumors. Endocr. Relat. Cancer.

[77] Krampitz G.W., George B.M., Willingham S.B., Volkmer J.P., Weiskopf K., Jahchan N., Newman A.M., Sahoo D., Zemek A.J., Yanovsky R.L. (2016). Identification of tumorigenic cells and therapeutic targets in pancreatic neuroendocrine tumors. Proc. Natl. Acad. Sci. USA.

[78] Imam R., Chang Q., Black M., Yu C., Cao W. (2021). CD47 expression and CD163(+) macrophages correlated with prognosis of pancreatic neuroendocrine tumor. BMC Cancer.

[79] Young K., Lawlor R.T., Ragulan C., Patil Y., Mafficini A., Bersani S., Antonello D., Mansfield D., Cingarlini S., Landoni L. (2021). Immune landscape, evolution, hypoxia-mediated viral mimicry pathways and therapeutic potential in molecular subtypes of pancreatic neuroendocrine tumours. Gut.

[80] Ono K., Shiozawa E., Ohike N., Fujii T., Shibata H., Kitajima T., Fujimasa K., Okamoto N., Kawaguchi Y., Nagumo T. (2018). Immunohistochemical CD73 expression status in gastrointestinal neuroendocrine neoplasms: A retrospective study of 136 patients. Oncol. Lett..

[81] Katsuta E., Tanaka S., Mogushi K., Shimada S., Akiyama Y., Aihara A., Matsumura S., Mitsunori Y., Ban D., Ochiai T. (2016). CD73 as a therapeutic target for pancreatic neuroendocrine tumor stem cells. Int. J. Oncol..

[82] Vesely C., Wong Y.N.S., Childs A., Akarca A.U., Dhami P., Vaikkinen H., Conde L., Herrero J., Ogunbiyi O., Gander A. (2022). Systematic Evaluation of the Immune Environment of Small Intestinal Neuroendocrine Tumours. Clin. Cancer Res..

[83] Apte R.S., Chen D.S., Ferrara N. (2019). VEGF in Signaling and Disease: Beyond Discovery and Development. Cell.

[84] Cigrovski Berkovic M., Cacev T., Catela Ivkovic T., Marout J., Ulamec M., Zjacic-Rotkvic V., Kapitanovic S. (2016). High VEGF serum values are associated with locoregional spread of gastroenteropancreatic neuroendocrine tumors (GEP-NETs). Mol. Cell. Endocrinol..

[85] Lin J.X., Weng X.F., Xie X.S., Lian N.Z., Qiu S.L., Wang J.B., Lu J., Chen Q.Y., Cao L.L., Lin M. (2019). CDK5RAP3 inhibits angiogenesis in gastric neuroendocrine carcinoma by modulating AKT/HIF-1alpha/VEGFA signaling. Cancer Cell Int..

[86] Luo X., He J.Y., Xu J., Hu S.Y., Mo B.H., Shu Q.X., Chen C., Gong Y.Z., Zhao X.L., Xie G.F. (2020). Vascular NRP2 triggers PNET angiogenesis by activating the SSH1-cofilin axis. Cell Biosci..

[87] Bosch F., Altendorf-Hofmann A., Jacob S., Auernhammer C.J., Spitzweg C., Boeck S., Schubert-Fritschle G., Werner J., Kirchner T., Angele M.K. (2020). Distinct Expression Patterns of VEGFR 1-3 in Gastroenteropancreatic Neuroendocrine Neoplasms: Supporting Clinical Relevance, but not a Prognostic Factor. J. Clin. Med..

[88] Zuazo-Gaztelu I., Paez-Ribes M., Carrasco P., Martin L., Soler A., Martinez-Lozano M., Pons R., Llena J., Palomero L., Graupera M. (2019). Antitumor Effects of Anti-Semaphorin 4D Antibody Unravel a Novel Proinvasive Mechanism of Vascular-Targeting Agents. Cancer Res..

[89] Keklikoglou I., Kadioglu E., Bissinger S., Langlois B., Bellotti A., Orend G., Ries C.H., De Palma M. (2018). Periostin Limits Tumor Response to VEGFA Inhibition. Cell Rep..

[90] Cavalcanti E., Ignazzi A., De Michele F., Caruso M.L. (2019). PDGFRalpha expression as a novel therapeutic marker in well-differentiated neuroendocrine tumors. Cancer Biol. Ther..

[91] Puliani G., Sesti F., Anastasi E., Verrico M., Tarsitano M.G., Feola T., Campolo F., Di Gioia C.R.T., Venneri M.A., Angeloni A. (2022). Angiogenic factors as prognostic markers in neuroendocrine neoplasms. Endocrine.

[92] Rigamonti N., Kadioglu E., Keklikoglou I., Wyser Rmili C., Leow C.C., De Palma M. (2014). Role of angiopoietin-2 in adaptive tumor resistance to VEGF signaling blockade. Cell Rep..

[93] Rodriguez-Remirez M., Del Puerto-Nevado L., Fernandez Acenero M.J., Ebrahimi-Nik H., Cruz-Ramos M., Garcia-Garcia L., Solanes S., Banos N., Molina-Roldan E., Garcia-Foncillas J. (2020). Strong Antitumor Activity of Bevacizumab and Aflibercept in Neuroendocrine Carcinomas: In-Depth Preclinical Study. Neuroendocrinology.

[94] Xu J., Shen L., Bai C., Wang W., Li J., Yu X., Li Z., Li E., Yuan X., Chi Y. (2020). Surufatinib in advanced pancreatic neuroendocrine tumours (SANET-p): A randomised, double-blind, placebo-controlled, phase 3 study. Lancet Oncol..

[95] Capdevila J., Fazio N., Lopez C., Teule A., Valle J.W., Tafuto S., Custodio A., Reed N., Raderer M., Grande E. (2021). Lenvatinib in Patients with Advanced Grade 1/2 Pancreatic and Gastrointestinal Neuroendocrine Tumors: Results of the Phase II TALENT Trial (GETNE1509). J. Clin. Oncol..

[96] Chang T.M., Chu P.Y., Hung W.C., Shan Y.S., Lin H.Y., Huang K.W., Chang J.S., Chen L.T., Tsai H.J. (2021). c-Myc promotes lymphatic metastasis of pancreatic neuroendocrine tumor through VEGFC upregulation. Cancer Sci..

[97] Wang Z., Peng L., Song Y.L., Xu S., Hua Z., Fang N., Zhai M., Liu H., Fang Q., Deng T. (2018). Pseudo-hemorrhagic region formation in pancreatic neuroendocrine tumors is a result of blood vessel dilation followed by endothelial cell detachment. Oncol. Lett..

[98] Massironi S., Facciotti F., Cavalcoli F., Amoroso C., Rausa E., Centonze G., Cribiu F.M., Invernizzi P., Milione M. (2022). Intratumor Microbiome in Neuroendocrine Neoplasms: A New Partner of Tumor Microenvironment? A Pilot Study. Cells.

[99] Hu W., Chen Z.M., Li X.X., Lu L., Yang G.H., Lei Z.X., You L.J., Cui X.B., Lu S.C., Zhai Z.Y. (2022). Faecal microbiome and metabolic signatures in rectal neuroendocrine tumors. Theranostics.

[100] Yachida S., Totoki Y., Noe M., Nakatani Y., Horie M., Kawasaki K., Nakamura H., Saito-Adachi M., Suzuki M., Takai E. (2022). Comprehensive Genomic Profiling of Neuroendocrine Carcinomas of the Gastrointestinal System. Cancer Discov..

[101] Bommareddy P.K., Shettigar M., Kaufman H.L. (2018). Integrating oncolytic viruses in combination cancer immunotherapy. Nat. Rev. Immunol..

[102] Leja J., Yu D., Nilsson B., Gedda L., Zieba A., Hakkarainen T., Akerstrom G., Oberg K., Giandomenico V., Essand M. (2011). Oncolytic adenovirus modified with somatostatin motifs for selective infection of neuroendocrine tumor cells. Gene Ther..

[103] Yu D., Jin C., Leja J., Majdalani N., Nilsson B., Eriksson F., Essand M. (2011). Adenovirus with hexon Tat-protein transduction domain modification exhibits increased therapeutic effect in experimental neuroblastoma and neuroendocrine tumors. J. Virol..

[104] Yamamoto Y., Nagasato M., Rin Y., Henmi M., Ino Y., Yachida S., Ohki R., Hiraoka N., Tagawa M., Aoki K. (2017). Strong antitumor efficacy of a pancreatic tumor-targeting oncolytic adenovirus for neuroendocrine tumors. Cancer Med..

[105] Kloker L.D., Berchtold S., Smirnow I., Schaller M., Fehrenbacher B., Krieg A., Sipos B., Lauer U.M. (2019). The Oncolytic Herpes Simplex Virus Talimogene Laherparepvec Shows Promising Efficacy in Neuroendocrine Cancer Cell Lines. Neuroendocrinology.

[106] Kloker L.D., Berchtold S., Smirnow I., Beil J., Krieg A., Sipos B., Lauer U.M. (2020). Oncolytic vaccinia virus GLV-1h68 exhibits profound antitumoral activities in cell lines originating from neuroendocrine neoplasms. BMC Cancer.

[107] Balkwill F.R., Capasso M., Hagemann T. (2012). The tumor microenvironment at a glance. J. Cell Sci..

[108] Zhou H., Wang Y., Guo C., Li X., Cui W., Wang Z., Chen X. (2021). Microscopic Invasion of Nerve Is Associated with Aggressive Behaviors in Pancreatic Neuroendocrine Tumors. Front. Oncol..

[109] Xu S.S., Xu H.X., Wang W.Q., Li S., Li H., Li T.J., Zhang W.H., Liu L., Yu X.J. (2019). Tumor-infiltrating platelets predict postoperative recurrence and survival in resectable pancreatic neuroendocrine tumor. World J. Gastroenterol..

[110] Werner R.A., Weich A., Higuchi T., Schmid J.S., Schirbel A., Lassmann M., Wild V., Rudelius M., Kudlich T., Herrmann K. (2017). Imaging of Chemokine Receptor 4 Expression in Neuroendocrine Tumors—A Triple Tracer Comparative Approach. Theranostics.

[111] Rodriguez Laval V., Pavel M., Steffen I.G., Baur A.D., Dilz L.M., Fischer C., Detjen K., Prasad V., Pascher A., Geisel D. (2018). Mesenteric Fibrosis in Midgut Neuroendocrine Tumors: Functionality and Radiological Features. Neuroendocrinology.

[112] Laskaratos F.M., Levi A., Schwach G., Pfragner R., Hall A., Xia D., von Stempel C., Bretherton J., Thanapirom K., Alexander S. (2021). Transcriptomic Profiling of In Vitro Tumor-Stromal Cell Paracrine Crosstalk Identifies Involvement of the Integrin Signaling Pathway in the Pathogenesis of Mesenteric Fibrosis in Human Small Intestinal Neuroendocrine Neoplasms. Front. Oncol..

[113] Daskalakis K., Karakatsanis A., Stalberg P., Norlen O., Hellman P. (2017). Clinical signs of fibrosis in small intestinal neuroendocrine tumours. Br. J. Surg..

[114] Laskaratos F.M., Rombouts K., Caplin M., Toumpanakis C., Thirlwell C., Mandair D. (2017). Neuroendocrine tumors and fibrosis: An unsolved mystery?. Cancer.

[115] Blazevic A., Zandee W.T., Franssen G.J.H., Hofland J., van Velthuysen M.F., Hofland L.J., Feelders R.A., de Herder W.W. (2018). Mesenteric fibrosis and palliative surgery in small intestinal neuroendocrine tumours. Endocr. Relat. Cancer.

[116] Bosch F., Bruewer K., D’Anastasi M., Ilhan H., Knoesel T., Pratschke S., Thomas M., Rentsch M., Guba M., Werner J. (2018). Neuroendocrine tumors of the small intestine causing a desmoplastic reaction of the mesentery are a more aggressive cohort. Surgery.

[117] Xu S., Xu H., Wang W., Li S., Li H., Li T., Zhang W., Yu X., Liu L. (2019). The role of collagen in cancer: From bench to bedside. J. Transl. Med..

[118] Huinen Z.R., Huijbers E.J.M., van Beijnum J.R., Nowak-Sliwinska P., Griffioen A.W. (2021). Anti-angiogenic agents—Overcoming tumour endothelial cell anergy and improving immunotherapy outcomes. Nat. Rev. Clin. Oncol..

[119] Pedersen K.S., Grierson P.M., Picus J., Lockhart A.C., Roth B.J., Liu J., Morton A., Chan E., Huffman J., Liang C. (2021). Vorolanib (X-82), an oral anti-VEGFR/PDGFR/CSF1R tyrosine kinase inhibitor, with everolimus in solid tumors: Results of a phase I study. Investig. New Drugs.

[120] Hobday T.J., Qin R., Reidy-Lagunes D., Moore M.J., Strosberg J., Kaubisch A., Shah M., Kindler H.L., Lenz H.J., Chen H. (2015). Multicenter Phase II Trial of Temsirolimus and Bevacizumab in Pancreatic Neuroendocrine Tumors. J. Clin. Oncol..

[121] Gile J.J., Liu A.J., McGarrah P.W., Eiring R.A., Hobday T.J., Starr J.S., Sonbol M.B., Halfdanarson T.R. (2021). Efficacy of Checkpoint Inhibitors in Neuroendocrine Neoplasms: Mayo Clinic Experience. Pancreas.

[122] Grande E., Rodriguez-Antona C., Lopez C., Alonso-Gordoa T., Benavent M., Capdevila J., Teule A., Custodio A., Sevilla I., Hernando J. (2021). Sunitinib and Evofosfamide (TH-302) in Systemic Treatment-Naive Patients with Grade 1/2 Metastatic Pancreatic Neuroendocrine Tumors: The GETNE-1408 Trial. Oncologist.

[123] Inoue M., Kim M., Inoue T., Tait M., Byrne T., Nitschke M., Murer P., Cha H., Subramanian A., De Silva N. (2022). Oncolytic vaccinia virus injected intravenously sensitizes pancreatic neuroendocrine tumors and metastases to immune checkpoint blockade. Mol. Ther. Oncolytics.

[124] Murphy T.L., Murphy K.M. (2022). Dendritic cells in cancer immunology. Cell. Mol. Immunol..

[125] Sharabi A., Tsokos M.G., Ding Y., Malek T.R., Klatzmann D., Tsokos G.C. (2018). Regulatory T cells in the treatment of disease. Nat. Rev. Drug Discov..

[126] Vitale G., Carra S., Ferrau F., Guadagno E., Faggiano A., Colao A., Nike (2020). Gastroenteropancreatic neuroendocrine neoplasms and inflammation: A complex cross-talk with relevant clinical implications. Crit Rev. Oncol. Hematol..

[127] Alvarez M.J., Subramaniam P.S., Tang L.H., Grunn A., Aburi M., Rieckhof G., Komissarova E.V., Hagan E.A., Bodei L., Clemons P.A. (2018). A precision oncology approach to the pharmacological targeting of mechanistic dependencies in neuroendocrine tumors. Nat. Genet.

[128] Weber E.W., Maus M.V., Mackall C.L. (2020). The Emerging Landscape of Immune Cell Therapies. Cell.

[129] Feng Z., He X., Zhang X., Wu Y., Xing B., Knowles A., Shan Q., Miller S., Hojnacki T., Ma J. (2022). Potent suppression of neuroendocrine tumors and gastrointestinal cancers by CDH17CAR T cells without toxicity to normal tissues. Nat. Cancer.

[130] Mu W., Chu Q., Liu Y., Zhang N. (2020). A Review on Nano-Based Drug Delivery System for Cancer Chemoimmunotherapy. Nano-Micro Lett..

[131] Longo S.K., Guo M.G., Ji A.L., Khavari P.A. (2021). Integrating single-cell and spatial transcriptomics to elucidate intercellular tissue dynamics. Nat. Rev. Genet.

[132] Yang K.C., Kalloger S.E., Aird J.J., Lee M.K.C., Rushton C., Mungall K.L., Mungall A.J., Gao D., Chow C., Xu J. (2021). Proteotranscriptomic classification and characterization of pancreatic neuroendocrine neoplasms. Cell Rep..

[133] Rindi G., Mete O., Uccella S., Basturk O., La Rosa S., Brosens L.A.A., Ezzat S., de Herder W.W., Klimstra D.S., Papotti M. (2022). Overview of the 2022 WHO Classification of Neuroendocrine Neoplasms. Endocr. Pathol..

